# Multidisciplinary Programed Learning Simulation to Improve Visual Blood Loss Estimation for Obstetric Trauma Scenarios

**DOI:** 10.30476/JAMP.2021.91990.1466

**Published:** 2022-01

**Authors:** JANE PONTERIO, MALEEHA AHMAD, APARNA VANCHESWARAN, NISHA LAKHI

**Affiliations:** 1 New York Medical College, 40 Sunshine Cottage Rd, Valhalla, NY 10591, United States; 2 Richmond University Medical Center, Department of Trauma Surgery and Obstetrics and Gynecology, 355 Bard Ave, Staten Island, NY, United States

**Keywords:** Simulation learning, Cesarean section, Obstetric

## Abstract

**Introduction:**

We designed and implemented a Programmed Learning Simulation (PLS) exercise depicting obstetric scenarios of hemorrhage to train anesthesiologists, ancillary staff, and surgeons to accurately estimate blood loss visually. We then measured the efficacy of this exercise in a clinical setting.

**Methods:**

We conducted a prospective study to assess the effect of implementing a PLS exercise on quantification of blood loss in an operative setting. The PLS exercise consisted of 13 simulation stations of varying quantities of simulated blood loss paired with standardized objects of known volume. Eighty-eight individuals participated including attending physicians, residents, medical students, and ancillary staff participated in this study. The PLS was part of regularly scheduled continuing medical education activities; thus, the sampling used was non-randomized convenience method. The percent error was calculated for each of the 13 stations. A subgroup analysis was performed to assess the effect of the years of experience, size of hemorrhage, and occupation on accuracy. Univariate analyses for continuous variables were compared using a one-way ANOVA test. For the comparison of the three groups (years of experience and size of hemorrhage), a p-value of <0.02 was considered statistically significant and for 5-way comparison (professional grouping) a p <0.01 was considered significant after application of the Bonferroni correction (α=0.05). (Part A). To determine the effect of PLS in a clinical setting, the percent error of blood loss estimation for cesarean deliveries during the two-month period after the PLS exercise was compared to the two-month period immediately prior to using the student’s t-test with p<0.05 as significant (Part B). Statistical analysis was performed using International Business Machine, Statistical Package for the Social Sciences, Version 26.0 (IBM SPSS).

**Results:**

During Part A, the baseline performance of the participants was evaluated during the PLS activity. The PLS data showed no significant difference in absolute value of mean percent error estimation (standard deviation) across professions: student 63.61% (69.74), ob/gyn 56.91% (47.72), ancillary 62.15% (77.90), general/trauma surgeon 66.70% (65.06), anesthesia 61.51% (63.12). (p = 0.681), or levels of experience 0-5: 62.21% (60.06), 6-10 years: 56.22% (52.66), greater than 10 years: 61.89% (71.89) (p = 0.831). However, mean percent error of estimation was higher when participants estimated smaller samples 77.7% (104.73) compared to either medium 56.8% (49.06) or large 57.9% (46.19) samples (p<0.001). For Part B, 179 cesarean deliveries occurred during the pre-intervention period and 193 occurred during the post-intervention period. Mean error in provider estimation of blood loss significantly improved from 47% (68.51) pre-intervention to 31% (32.70) post-intervention (p=0.009).

**Conclusion:**

We believe our described PLS activity was effective in teaching techniques for visual blood loss estimation. This was reflected by improved competency in a clinical setting, demonstrated by more accurate visually estimated blood loss during the period immediately following simulation activity compared to a prior time frame. Further research is needed to assess the impact of simulation activities on patient outcomes, such as utilization of blood products and patient morbidity.

## Introduction

Trauma complicates 6-7% of all pregnancies and is the leading cause of non-obstetric maternal morbidity and mortality. An analysis conducted by WHO reported about 73% of all maternal mortalities worldwide were attributed to true obstetric etiologies with hemorrhage as the leading cause of death ( 1 ). Identification of hypovolemic shock during pregnancy poses challenges due to the unique physiological adaptions, including increased plasma volume, stroke volume, and cardiac output ( 2 ). Shock may not manifest until the total blood volume depletion reaches 30-35%, as compared to 15% in a non-pregnant patient ( 3 - 5 ). Clinicians are unable to rely on vital signs, physiological changes, or indications of end organ damage until patients are in a critical condition. The use of vital signs alone is impractical. Thus, accurate estimation of cumulative blood loss can forewarn impending hemodynamic compromise. 

Estimation methods are non-standardized, but a few commonly used techniques include visual, gravimetric, and colorimetric methods. The visual method involves estimation by the clinician through visual assessment of blood contaminated surgical equipment like surgical sponges and suction containers. Gravimetric methods involve subtracting blood-soaked surgical equipment from their dry weights to indirectly measure the blood loss. This value is then combined with the amount of liquid present in collection containers ( 6 ). Lastly, colorimetric estimation uses technology platforms to calculate the blood loss from photographs of soaked surgical materials. The algorithm filters out non-blood, colored components in the photographs through colorimetric analysis and determines hemoglobin mass present ( 6 , 7 ). Clinically, during simulation, quantitative methods like gravimetric and colorimetric have been shown to improve blood loss estimation ( 7 - 12 ). For instance, Lilley et al. showed gravimetric estimation of blood loss offered a significantly lower mean percent error (4.0±2.7%) in estimation compared to visual estimation (34.7±32.1%) ( 12 ). Gerdessen et al. performed a meta-analysis which compared modalities of blood loss estimation and found that colorimetric techniques offered the highest degree of accuracy in blood loss estimation. This is supported by another study which reported visual and gravimetric methods having a higher degree of bias when compared to colorimetric ones ( 7 ). Medical literature attempts to prove one modality superior to another; however, these studies show conflicting results and state the evidence to be insufficient ( 13 ). In addition, each method possesses its own flaws for measurement and is vulnerable to inaccuracy ( 7 ). For instance, the addition of amniotic fluid in obstetric procedures as well as external blood loss in trauma situations can confound gravimetric analysis. A major barrier to implementation of colorimetric analysis is access to an artificial-intelligence technology.

Historically, visual estimation is the most practical method of measurement, especially in obstetric emergencies, where quantitative measures cannot feasibly be applied due to time and physical constraints ( 13 - 15 ). However, the inaccuracy of visual blood loss assessment has been illustrated in several studies ( 8 , 16 - 18 ). Stafford et al. compared the accuracy of visually estimated blood loss (EBL) to calculate blood loss (CBL) for both vaginal and cesarean deliveries. The results found EBL to be significantly inaccurate when compared to CBL for both modes of delivery ( 18 ). These findings remain true in a recent simulation study that showed inaccuracies in visual EBL in a video series of emergent and non-emergent injuries ( 19 ). Most studies have shown that at lower volumes visual estimation has a tendency to overestimate blood loss ( 8 , 19 ). On the other end, at higher volumes, visual estimation tends to underestimate blood loss ( 4 , 20 ). Supporting this tendency of underestimation, Lertbunnaphong et al. found visual techniques underestimated blood loss compared to the gravimetric drape method, as the visual method missed 65.4% of post-partum hemorrhage diagnoses ( 20 ). Another supporting study reported that the participants overestimated smaller blood volumes between 50ml to 200ml and underestimated blood volumes greater than 400ml. This study also reported a greater percent error in estimation as blood volumes increased ( 19 ). One study disapproved the inaccuracy of the visual method and showed it to be equal to CBL when approximating the volumes <500mL ( 21 ). Additional factors such as professional experience and training show conflicting results, with some showing no improvement in accuracy ( 10 , 22 , 23 ). Regardless of these shortcomings, the decision to transfuse in obstetric emergencies has shown no significant difference between various modalities of blood loss estimation ( 9 , 24 , 25 ). 

Our simulation is novel as it utilizes a programmed learning didactic approach. Programmed Learning/Instruction is the process of arranging the material to be learned into a series of sequential steps to help the learner form mental associations between familial and unfamiliar concepts. As an educational technique, the learner is presented a logical sequence of materials, with multiple content repetitions. Immediate feedback is given, which serves as reinforcement of the content. 

A systematized method of blood loss estimation may increase awareness that a massive obstetric hemorrhage has occurred, thereby allowing for earlier intervention and improved communications during trauma situations and transition of care. Thus, the objective of this study was to design and implement a simulation exercise depicting obstetric scenarios of hemorrhage to train anesthesiologists, ancillary staff, and surgeons to estimate blood loss visually more accurately. We hypothesized that a programmed learning didactic simulation exercise would improve the provider’s estimation of blood loss in a clinical setting. In order to test this hypothesis, we compared the percent error of blood loss estimation for cesarean deliveries after the simulation exercise to the period immediately prior. 

## Methods

### Part A: The Programmed Learning Simulation

We conducted a prospective study to assess the effect of implementing a PLS exercise on quantification of blood loss in an operation setting. This study was reviewed by the Institutional Review Board of our affiliated university, New York Medical College, Valhalla, New York, and received an exemption. The PLS was part of regularly scheduled continuing medical education activities, thus the sampling was a non-randomized convenience method. The programmed learning simulation (PLS) exercise was administered to 88 learners consisting of 24 third-year medical students, 19 obstetrics and gynecology physicians, 22 ancillary staff members (operation room nurses and technicians), 10 trauma surgeons, and 14 anesthesiologists at Richmond University Medical Center, Staten Island, New York. For the residents and attending physicians, participation was mandatory as part of their continuing medical education. Nurses, ancillary staff, and medical students were highly encouraged to participate and did so voluntarily. The participants were told that their answers may be used for quality improvement/research purposes; however, there was no formal consent process. 

The PLS was conducted jointly by attending physician leaders in the ob/gyn and trauma surgery departments. The didactic activity took place over the course of three sessions spanning a ten-day time period. For each session, the stations were recreated with fresh artificial blood to avoid spoilage. Participants were expected to maintain confidentiality and requested not to share information regarding the simulations or answers to the simulation station estimates with their colleagues. 

The PLS exercise was designed to teach the providers how to visually quantify blood loss using a series of thirteen simulation stations. Each station depicted a simulated amount of blood loss for a clinical scenario. For this PLS exercise, artificial blood was prepared using non-validated mixture light corn syrup, water, and red food coloring with the first two ingredients in a 5:1 ratio and drops of coloring added until the desired hue was achieved. However, several more contemporary recipes are available based on the desired quantity and consistency ( 26 ). All operating room supplies were obtained from the labor and delivery floor of Richmond University Medical Center. 

The ninety-minute PLS exercise was executed in two parts, followed by a debriefing. For Part 1 (twenty-six minutes), the participants visited each of the thirteen stations (two minutes each) and were asked to visually quantify the amount of simulated blood loss depicted. They solely used visual cues such as the number of soaked laps, appearance of operation field, etc.; however, they were not permitted to touch or move any of the objects. The thirteen stations were arranged in a circuit design. Two to three participants were positioned at each of the thirteen stations along the circuit. The participants were instructed to work individually and not to communicate the answers with each other. After the two-minute period elapsed, an alarm was sounded, and participants were instructed to move to the next station along the circuit loop, while at each station, participants documented their answers on a response sheet that was collected at the end of Part 1. The response sheet solicited demographic information (department, level of experience) and had thirteen blank numbered lines for free form documentation of answers to each simulation station. During Part 1 of the PLS activity, each simulation station was viewed without its corresponding paired object.

Following Part 1 and after collection of the response sheets, paired objects along with a card revealing the correct answer were placed at each station. This commenced the transition to Part 2 (thirteen minutes) where participants, in groups or two-three people, revisited each of the thirteen stations for an additional one minute. Participants were free to discuss the station, paired objectives, and original answers among the group members at this time. Like Part 1, an alarm bell was sounded to signal when it was time to move to the next station along the circuit. The final debriefing session (approximately fifty minutes) consisted of open discussion and didactic teaching session. The debriefing was conducted by physician leaders in the ob/gyn department and trauma surgery departments who facilitated the simulation. During this session, the answers to each of the thirteen simulations were discussed. Additionally, formal techniques for visually estimating blood loss were presented to the participants ( 6 ). Each person’s performance was measured by scoring of the response sheet that was collected at the end of Part 1. By providing immediate feedback to the learner during the debriefing session, we were consistent in maintaining the core tenets of programmed learning simulation. 

### Statistical Analysis for Part A

All statistical analyses were carried out using International Business Machine, Statistical Package for the Social Sciences, Version 26.0 (IBM SPSS). In order to assess normality, data points were plotted and observed. Outliers were not excluded from analysis as these extreme data points reflect trends in gross overestimation or underestimation of blood loss is well documented in the literature ( 4 , 8 , 19 - 20 ). We utilized both percent error and absolute value of percent error as they account for different aspects of error in estimation. The percent error calculation accounts for bidirectional error in either overestimation or underestimation. However, it does not account for the magnitude of error as the positive values cancel the negative ones. Thus, we also report the absolute value of the percent error as it accounts for the absolute magnitude of error in either direction. The percent error of estimated blood loss was calculated for each participant response using the

Following formula ( 17 ): 

$\mathrm{\% Error for blood estimation}=\frac{\left(\mathrm{Estimated Blood Volume}-\mathrm{Actual Blood Volume}\right)}{\mathrm{Actual Blood Volume}}\times 100$

Descriptive statistical analysis of percent error (mean, range) was calculated for each of the thirteen stations. The absolute value of percent error estimation was calculated by taking the absolute value of the percent error calculation. A pooled subgroup analysis for all thirteen stations was performed to assess the inﬂuence of years of experience (0-5 years, 6-10 years, and greater than 10 years), size of hemorrhage (Small: 30 ml, 60 ml, and 100 ml; Medium: 150 ml, 250 ml, and 500 ml; Large: 1000 ml, 1500 ml, and 2000 ml), and occupational status (medical student, resident, obstetric surgeon, general/trauma surgeon, anesthesiologist, and ancillary staff- OR nurses or technicians) on the accuracy of estimation. Univariate analyses for continuous variables were compared using one-way ANOVA. In order to minimize the chance of making at Type 1 error when making multiple group comparisons, we applied a Bonferroni correction (α_new_ = α_original_ / number of groups) to an alpha original of 0.05. Thus, for the three group comparisons (years of experience and size of hemorrhage), a p-value of <0.02 (0.05/3) was considered statistically significant and for 5-way comparison (professional grouping) a p <0.01 (0.05/5) was considered significant after application of a Bonferroni correction. When differences between three or more groups were statistically significant, post-hoc analysis was performed to detect a significant differences between the groups. 

### Part B: Effect of the PLS in a Clinical Setting

In order to test the effect of the PLS activity in a clinical setting, we compared the error of blood loss estimation for cesarean deliveries after the simulation exercise to the period immediately prior. Cesarean deliveries performed during the two-month period prior to the simulation exercise were included in the pre-interventional group and cesarean deliveries that occurred in the two-month period after the simulation exercise were included in the post-interventional group. Exclusion criteria were emergency cesarean deliveries (fetal distress, placental abruption, cord prolapse etc.), patients transfused with blood products intraoperatively or post-operatively during the first 24 hours, or those patients that refused a post-operative Complete Blood Count (CBC). A de-identified database grouped by the month when the cesarean sections occurred was maintained. 

At our institution, it is routinely practiced for the estimated blood loss (EBL) for each cesarean delivery to be determined by an informal joint consensus of the attending surgeon, resident, and anesthesiologist. The final documentation EBL is done by the resident in the medical record.  This practice was the same both prior to and after the PLS. The staff were not told that their EBL values were being monitored by the obstetric department after the PLS activity to avoid observational bias. The participants in Part B included the 19 ob/gyn physicians, 22 ancillary staff members, and 14 anesthesiologists who took part in the PLS exercise. The calculated blood loss (CBL) was obtained by multiplying the maternal blood volume by the percent change in hematocrit ( 18 ):

Calculated Blood Loss = (Maternal Blood Volume) x (% change in hematocrit), 

Where the maternal blood volume was determined for a standardized formula ( 18 ): 

**Maternal Blood Volume** = 0.75 x [(maternal height in inches x 50) + (maternal weight in pounds x 25)] 

and the percent change in hematocrit was calculated from the following formula ( 17 ): 

$\mathrm{\% change in hematocrit}=\frac{\left(\mathrm{pre delivery hematocrit}-\mathrm{post-delivery hematocrit}\right)}{\mathrm{pre delivery hematocrit}}$

The pre-delivery CBC, used to assess the hematocrit, was obtained at admission to labor and delivery, prior to the cesarean delivery. The post-operative CBC was performed the morning after the index cesarean delivery, as done routinely at our institution on all post-cesarean patients. Maternal weight and height were determined at admission to Labor and Delivery.

The percent error in EBL was calculated using the CBL as a basis of comparison yielding a percent error value ( 17 ):

$\mathrm{\% Error for blood estimation}=\frac{\left(\mathrm{Estimated Blood Loss}-\mathrm{Calculated Blood Loss}\right)}{\mathrm{Calculated Blood Loss}}\times 100$

### Statistical Analysis for Part B

Descriptive statistical analysis of percent error (mean, range) was calculated for each pre- and post-interventional period. The absolute value of percent error estimation was calculated by taking the absolute value of the percent error calculation. Comparison between the pre- and post-intervention data sets was analyzed using student’s t-test. A p-value <0.05 was considered statistically significant. 

## Results

### Part A

A total of 88 clinicians, consisting of 24 medical students, 19 ob/gyn physicians, 22 ancillary staff members, 10 surgeons, and 14 anesthesiologists participated in the PLS exercise. Years of experience among providers were: 0-5 years (n=39), 6-10 years (n=9), and greater than 10 years (n=40). Table 1 depicts mean percent error and absolute value of mean percent error for all the thirteen stations. The results of each station in Table 1 are presented as a mean aggregate of all learners that participated in the simulation.

**Table 1:** Mean Percent Error of EBL by Station

Station #	Artificial Blood Volume (ml)	Mean Estimate, ml (Range)	Mean % Error (Range)	Absolute Value of Mean % Error (Standard Deviation)
Station 1	100	120 (10 – 500)	19.94 (-90 – 400)	68.13 (87.12)
Station 2	30	53 (5 – 250)	75.76 (-83.33 – 733.33)	110.98 (150.71)
Station 3	60	41 (5 – 150)	-30.97 (-91.67 – 150)	53.88 (32.70)
Station 4	150	139 (20 – 500)	-7.42 (-86.67 – 233.33)	54.55 (38.30)
Station 5	1,500	603 (50 – 2,500)	-59.81 (-96.67 – 66.67)	63.60 (24.87)
Station 6	250	206 (20 – 800)	-17.55 (-92 – 220)	60.70 (40.49)
Station 7	1,000	1,495 (70 – 3,500)	45.94 (-93 – 250)	70.24 (66.80)
Station 8	2,000	1,781 (100 – 4,500)	-10.93 (-95 – 125)	39.84 (28.88)
Station 9	500	365 (50 – 2,000)	-27.07 (-90 – 300)	51.84 (41.73)
Station 10	250	267 (50 – 1,500)	6.70 (-80 – 500)	54.11 (65.02)
Station 11	500	226 (20 – 1,000)	-54.86 (-96 – 100)	57.59 (25.68)
Station 12	150	183 (20 – 900)	21.67 (-86.67 – 500)	68.18 (75.72)
Station 13	150	349 (20 – 1,500)	-30.26 (-96 – 200)	50.40 (36.05)

EBL: Estimated Blood Loss

The mean and range of percent error, which represent the range of error in over- and underestimation, are shown in the first column of Table 2. The significant p-values indicate that the mean estimates differ from one another; however it does not indicate that one group was more accurate at estimation than another. For example, the medical students mean percent error was -0.44, which seems better than the ob/gyn physicians who had a mean percent error of -29.95. However, the range of error for medical students was much broader, accounting for the result. Thus, there are limitations to solely relying on the mean percent error calculation. The most notable limitation is that it does not account for the actual magnitude of error as the positive values (overestimation) cancel the negative ones (underestimation). Thus, we also report the absolute value o the mean percent error, a metric which accounts for the magnitude of error regardless of over- or under-estimation.

**Table 2:** Mean % Error of EBL by Subgroup

Subgroup	Mean % Error (Range)	p *	Absolute Value of Mean % Error (Standard Deviation)	p
Professional grouping (N)
Student (24)	-0.44 (-96.00 – 733.33)	<0.001	63.61(69.74)	0.681
OB/Gyn (19)	-26.95 (-95.00 – 500)		56.91(47.72)	
Ancillary (22)	14.86 (-87.50 – 566.67)		62.15 (77.09)	
Surgeons (10)	-14.03 (-96.00 – 566.67)		66.70 (65.06)	
Anesthesia (14)	-8.38 (-96.67 – 566.67)		61.51 (63.12)	
Years of experience (N)
< 5 years (39)	-15.11 (-96.33 – 733.33)	0.004	62.21 (66.06)	0.831
6-10 years (9)	11.46 (-80.00 – 233.33)		56.22 (52.66)	
> 10 years (40)	1.62 (-96.67 – 250.00)		61.98 (71.89)	
Blood Loss Sample Size
Small: 30, 60, and 100mL	21.58 (-91.67 – 733.33)	<0.001	77.66 (104.73)	<0.001*
Medium: 150, 250, and 500mL	-15.55 (-96.00 – 500.00)		56.77 (49.06)	
Large: 1000, 1500, and 2000mL	-8.07 (-96.67– 250.00)		57.87 (46.19)	

EBL: Estimated Blood Loss; p-value (Absolute Value Mean % Error) small vs. medium: 0.006; small vs. large: 0.014; medium vs. large: 0.946

* For mean percent error, the significant p-values indicate that the mean estimates differ from one another; however, it does not indicate one group was more accurate at estimation than another. Thus, post hoc calculations were not performed.

**Figure 1:**
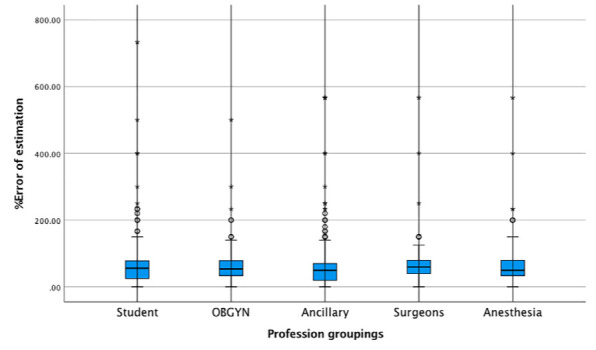
Percent Error of Estimated Blood Loss Across Professional Groupings

**Figure 2:**
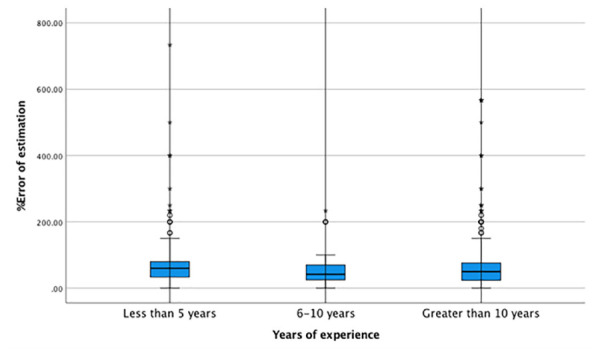
Percent Error of Estimated Blood Loss Across Levels of Experience

**Figure 3:**
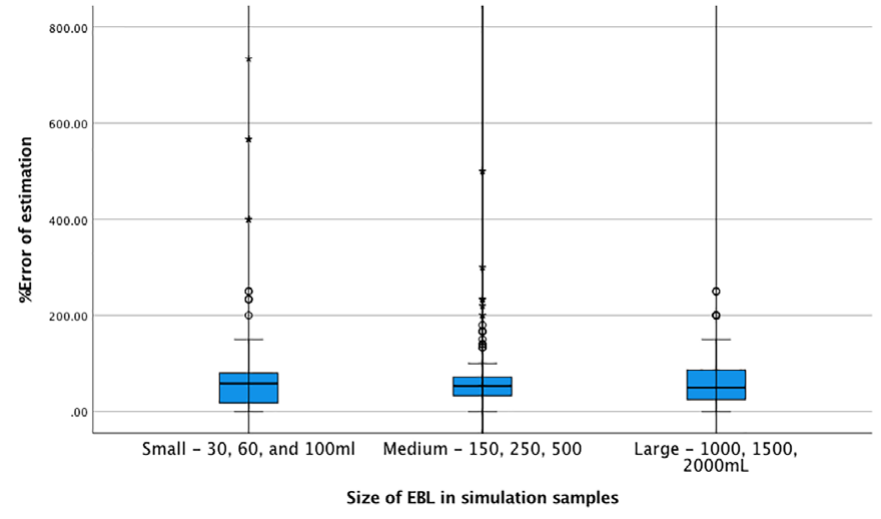
Percent Error of Estimated Blood Loss for Small, Medium, and Large Volumes

The absolute value of mean percent error (standard deviation) by the provider group was similar (p=0.681): student 63.61% (69.74), ob/gyn 56.91% (47.72), ancillary 62.15% (77.90), general/trauma surgeons 66.70% (65.06), and anesthesia 61.51% (63.12) ( Table 2). This non-significant difference in absolute percent error indicates a similar magnitude of error estimation regardless of professional background. Furthermore, varying levels of experience in the participants’ respective fields yielded no statistically significant difference in the absolute values of percent error estimation (p=0.831). The absolute value of the mean percent error for those with 0-5 years was 62.21% (60.06), 6-10 years 56.22% (52.66), and greater than 10 years 61.89% (71.89) ( Table 2). There were, however, differences in how accurately the providers estimated small samples of blood loss as compared to medium or large samples. The absolute values of mean error of the provider estimated blood loss for small samples was larger, 77.67% (104.73), as opposed to 56.77% (49.06) for medium samples and 57.87% (46.19) for large samples (p<0.001) ( Table 2). The box-and-whisker plots in Figure 1-3 visually depict the mean absolute error and percent error in estimation, absolute range of error estimates, interquartile range (blue box), all outliers for the professional groupings (2A), years of experience (2B), and volume sizes (2C).

### Part B

There were 179 cesarean procedures (86 primary, 93 repeat) that met the inclusion criteria during the two-month pre-interventional period and 193 (105 primary, 88 repeat) during the two-month post-intervention period. The providers’ errors in blood loss were compared between the two periods. 

The mean percent error (standard deviation), which accounts for bidirectional error in either over-estimation or under-estimation, was reduced from 23.4% (79.31) pre-intervention to 10.6% (44.17) post-intervention; however, this reduction just fell short of statistical significance( p-value 0.053). ( Table 3) The absolute value of mean error (standard deviation) in provider-estimated blood loss was significantly reduced in the post-interventional period to 31.5% (32.69), compared to 47.2% (68.51) pre-intervention (p=0.009), indicating a statistically significant improvement in post-intervention accuracy of blood loss estimation. 

**Table 3:** Clinical Data Pre- and Post-PLS Simulation Activity

	Pre-PLS Activity	Post-PLS Activity	p
Calculated Blood Loss (ml) Mean (Range)	807.4 (110.3 – 1862.7)	829.6 (282.9 – 1945.1)	0.507
Estimated Blood Loss (ml) Mean (Range)	803.9 (500 – 2000)	818.9 (600 – 3500)	0.468
Percent Error Mean (Range)	23.4 (-66.1 – 625.3)	10.6 (-57.6 – 182.8)	0.053
Absolute Value of Percent Error Mean (Range)	46.2 (0.7 – 625.3)	31.5 (0.04 – 182.8)	0.008

PLS: Programmed Learning Simulation

Table 3 shows comparison of pre- and post-simulation calculated blood loss (mean, range), estimated blood loss (mean, range), percent error (mean, range), and absolute value of mean percent error (mean, range). 

## Discussion

The importance of quantifying the amount of blood loss associated with obstetric hemorrhage should not be undervalued. Due to physiological changes associated with pregnancy, signs of hemodynamic instability such as tachycardia and hypotension do not manifest initially ( 2 , 3 ). Although colorimetric and gravimetric techniques of blood loss estimation may be utilized, weighing of materials and using gravimetric measurements are time consuming and not always practical in clinical and trauma situations ( 7 , 13 - 15 ). By designing a simulation exercise to teach providers techniques in visual estimation of blood loss, we sought to improve recognition of massive blood loss that can occur in intraoperative and obstetrical trauma situations. Early recognition of a massive hemorrhage may improve the patient outcomes by prompting the providers to initiate morbidity-reducing interventions, such as prolonged observation in a recovery room or immediate transfusion of blood products. 

Programmed Learning/Instruction is the process of arranging the material to be learned into a series of sequential steps to form the associations between familial and unfamiliar concepts. Programmed Learning is a term derived from the tenants of operant or conditioned learning. It was first proposed by B.F. Skinner of Harvard University in the 1940’s in a paper entitled ‘Science of Learning and art of Teaching’ ( 27 ). In this paper, Skinner argued that the desirable behavior can be brought out by continuous feedback to the learner. Our programed learning simulation, inspired by this model of teaching, was designed in two steps. During Part 1, learners were exposed to simulated volumes of blood loss and asked to visually quantify it. During Part 2, familiar objects of known volumes were paired with each simulation station. By using known familiar objects, we sought to program the association between the object and the simulated volume visualized. Although our study was not designed to test this hypothesis, we felt that the use of a concrete object helped our learners quantify abstract volumes. For example, some learners may struggle, if asked, to mentally conceptualize 750ml of blood; however, most can easily picture a bottle of wine, which also contains 750ml of fluid. Our programed learning approach builds upon this concept of association. 

Majority of prior studies conducted to determine the impact of didactic training on blood loss assessment have demonstrated a very short-term increase in accuracy post training. Dildy et al. presented a twenty-minute Power Point presentation where techniques to estimate blood loss were discussed ( 28 ). Pre- and post-test using seven clinical reconstructions were administered to fifty-three clinical providers. Median percent error in blood loss estimation was significantly reduced for all seven clinical reconstructions post-didactic session. Similarly, a study by Poolnoi et al. used pictograms of measured blood volumes designed to educate providers on techniques of blood loss estimation.  Forty-nine providers participated in pre- and post-tests where they were given pictures from cesarean sections and asked to estimate the blood volume depicted. After the didactic activity, the accuracy of estimation increased significantly from 30.9% to 61.8% ( 29 ). Sukprasert et al. randomized ninety obstetric nurses into two groups: one receiving a didactic course on blood loss estimation and the other received no instruction ( 30 ). The nurses that received the educational session had a significantly greater percent accuracy on estimating blood loss as assessed by a post-test clinical reconstruction compared to the nurses who received no instruction. 

Unlike many of the previous studies which tested the impact of a didactic session using an immediate post-test as the endpoint, our study assessed the effect of a didactic session in clinical practice over a prolonged two-month period. A study by Toledo et al. also assessed longitudinal retention of learning using web-based simulations of blood loss. At nine-months follow-up, the median percent error in estimation declined from -13.5% immediately post-training to -34.6% ( 31 ). Similarly, the Illinois Obstetric Hemorrhage Project mandated all obstetric service providers in the State of Illinois, to present a didactic activity on blood loss estimation consisting of a pre-test, didactic lecture, skill stations, and debriefing. Pooled data from 95 hospitals (n=9,456) indicated improvement in estimation, with participants scoring above 88% correct answers increasing from 10.9% on the pre-test to 49.1% at 6-month follow-up ( 32 ). Although both these studies measure longitudinal retention of learning using a post-test, we used a clinical endpoint to test the effectiveness of our didactic simulation exercise over time. For this clinical endpoint, we decided to use cesarean delivery as our clinical representation of obstetric hemorrhage because it is standardized and has occurred frequently. 

This, however, was not without limitations. First, the formula used to derive calculated blood loss relied on the differences between pre- and post-operative hematocrit measurements. Whilst it is a standard practice to collect this information on each patient at our hospital, the formula does not account for post-operative bleeding and fluid replacement. We attempted to compensate for it by excluding patients that required blood transfusion within 24 hours of delivery. Second, on average there was at least one person at each cesarean delivery who attended the PLS training. This was due to logistic reasons (holidays, maternity leave, off-duty hours, etc.) and not every resident and attending physician attended the PLS activity. We suggest that other hospitals implementing similar didactic activities schedule multiple sessions throughout the calendar year to expand attendance. In spite of these limitations, the initial results of this study are encouraging as it demonstrated that our programmed learning simulation exercise improved the providers’ accuracy of blood loss estimation, and this improvement persisted for two months after the intervention. 

Several studies have shown overestimation at low volumes ( 8 , 19 ) and underestimation a high volume ( 4 , 20 ), both of which can have clinical implications. Whilst underestimation at high volumes can lead to the heavy obstetric hemorrhage being unrecognized, underestimation at low volumes may trigger over transfusion of blood products and increase both patients’ stress and provider’s anxiety. Interestingly, in our study, we saw a trend towards underestimation at low volumes and overestimation at medium and high blood volumes (mean percent error, Table 2). The largest magnitude of error occurred at small volume blood loss (absolute value percent error, Table 2). Our groupings of small, medium, and large were based upon the chosen volumes of the thirteen simulated stations. For learning purposes, some of the volumes were repeated. We felt presenting the same volume in different simulated scenarios would aid in learning, visualization, and retention as they may appear differently in unique situations. Additionally, given the similarities between the higher volume in the small group (100 ml) and smaller volumes in the medium group (150 ml and 250 ml), some overlaps might have occurred, limiting final conclusions in this area. Outliers were not excluded from analysis as we felt that these data points were legitimate as significant over- and under-estimation of blood loss is well documented in the literature ( 4 , 8 , 19 - 20 ).

Although one may assume more experienced clinicians to have improved accuracy compared to novice clinicians, the literature is conflicting. Similar to some studies, we found no difference in accuracy between the provider groups or increasing years of experience ( 10 , 22 , 23 ). Rothermel and Lipman compared the accuracy of visually estimated blood loss across different specialties, years of experience, and levels of confidence in assessment. The study demonstrated that when presented with operative simulations of blood loss in varying magnitudes, surgeons, anesthesia providers, nurses, and technicians demonstrated similar levels of inaccuracy in visual estimation. There was also no association between increasing years of experience or confidence in ability to quantify blood loss with respect to accuracy of estimation ( 33 ). However, Dildy et al. found a higher likelihood of blood loss underestimation in cesarean deliveries in professionals with longer years of experience ( 28 ). Given that visual blood loss estimation is an abstract concept, years of clinical practice or professional experience may not be sufficient to improve their skill. Of interest, with regard to provider experience, the "6-10 years of experience" group only had 9 participants, while the other two groups averaged around 40. This was due to the large number of medical students/residents and senior attendings at our institution. This strengthens the argument that didactic instruction with assessment and feedback is needed for providers at all levels of experience.Our study did not collect data on clinical outcomes such as time to activation of transfusion protocols and utilization of blood products pre- and post-PLS. One study that compared the use of quantitative blood loss (QBL) methods to visual EBL in post-partum hemorrhages (PPH) showed similar outcomes for the use of blood transfusion, i.e. 2.7% and 2%, respectively ( 34 ). Another study which compared EBL and QBL to predict the need for postpartum blood transfusion also showed no difference with QBL reporting higher rates of blood loss >1000ml ( 24 ). The ability to visually estimate blood volumes to guide management, especially when initiating blood saving products, is an important skill in trauma and surgery. Further research on the effect of simulation training on clinical outcomes is necessary. 

Strengths of our PLS include use of a programmed teaching approach that paired familiar objects with specified hemorrhage simulations.  Additionally, our PLS was administered across multiple specialties and varying skill levels. We chose a clinical end point, cesarean delivery, to test the effect of our PLS and found improvement in estimation. At our institution, it has always been a routine practice for providers to informally discuss blood loss and come to an agreed estimate. Thus, conditions were similar pre- and post-PLS. Moreover, after the PLS was conducted, providers were not told that their estimates were being monitored, eliminating observational bias. However, there were limitations to choosing cesarean delivery as an endpoint as discussed previously. Additional limitations include non-standardized groupings of small, medium, and large objects, and small number of stations utilizing surgical equipment (gauzes, laps, chux, etc.). However, simulation stations can be customized to the group of learners depending on the concepts and skills being taught. 

## Conclusion

Visual estimation of blood loss is a teachable skill. We believe our described PLS activity was effective in teaching providers techniques for visual blood loss estimation. Our results demonstrated improved competency in a clinical setting, with more accurate visually estimated blood loss immediately following the simulation activity compared to pre-intervention. 

Future research relating to visual blood loss estimation is needed to improve clinical practice. Studying the longevity of simulation training will help decide the frequency of simulation labs to ensure retention and improve clinical outcomes. Additionally, further studies are necessary to assess the impact of simulation activities on patient outcomes such as utilization of blood products and patient morbidity. 

## Acknowledgement

We would like to acknowledge the Trauma Department for their collaboration. Additionally, we would like to thank Dr. Michael Moretti for his initial conception of programed learning simulations and Dr. Adaya Sharma for helping to set up the simulation stations and making the artificial blood.

**Conflict of Interest:** None Declared. 

## References

[1] Say L, Chou D, Gemmill A, Tunçalp Ö, Moller AB, Daniels J, et al ( 2014). Global causes of maternal death: a WHO systematic analysis. Lancet Glob Health.

[2] Soma-Pillay P, Nelson-Piercy C, Tolppanen H, Mebazaa A ( 2016). Physiological changes in pregnancy. Cardiovasc J Afr.

[3] Bonanno FG ( 2012). Hemorrhagic shock: The "physiology approach". J Emerg Trauma Shock.

[4] Patel A, Goudar SS, Geller SE, Kodkany BS, Edlavitch SA, Wagh K, et al ( 2006). Drape estimation vs. visual assessment for estimating postpartum hemorrhage. Int J Gynaecol Obstet.

[5] Hooper N, Armstrong TJ Hemorrhagic Shock [Updated 2020 Nov 21]. In: StatPearls [Internet]. Treasure Island (FL): StatPearls Publishing; 2021 Jan. https://www.ncbi.nlm.nih.gov/books/NBK470382/..

[6] Quantitative Blood Loss in Obstetric Hemorrhage [Internet] ACOG. https://www.acog.org/clinical/clinical-guidance/committee-opinion/articles/2019/12/quantitative-blood-loss-in-obstetric-hemorrhage.

[7] Gerdessen L, Meybohm P, Choorapoikayil S, Herrmann E, Taeuber I, Neef V, et al ( 2021). Comparison of common perioperative blood loss estimation techniques: a systematic review and meta-analysis. J Clin Monit Comput.

[8] Natrella M, Di Naro  E, Loverro M, Benshalom-Tirosh N, Trojano G, Tirosh D, et al ( 2018). The more you lose the more you miss; accuracy of postpartum blood loss visual estimation: A systematic review of the literature. J Matern Fetal Neonatal Med.

[9] Rubenstein AF, Zamudio S, Al-Khan A, Douglas C, Sledge S, Tully G, et al ( 2018). Clinical experience with the implementation of accurate measurement of blood loss during cesarean delivery: influences on hemorrhage recognition and allogeneic transfusion. Am J Perinatol.

[10] Toledo P, McCarthy RJ, Hewlett BJ, Fitzgerald PC, Wong CA ( 2007). The accuracy of blood loss estimation after simulated vaginal delivery. Anesth Analg.

[11] Razvi K, Chua S, Arulkumaran S, Ratnam SS ( 1996). A comparison between visual estimation and laboratory determination of blood loss during the third stage of labour. Aust N Z J Obstet Gynaecol.

[12] Lilley G, Burkett-St-Laurent D, Precious E, Bruynseels D, Kaye A, Sanders J, et al ( 2015). Measurement of blood loss during postpartum haemorrhage. Int J Obstet Anesth.

[13] Diaz V, Abalos E, Carroli G ( 2018). Methods for blood loss estimation after vaginal birth. Cochrane Database Syst Rev.

[14] Hill CC, Pickinpaugh J ( 2008). Trauma and surgical emergencies in the obstetric patient, Surgical Clinics of North America. Surg Clin North Am.

[15] Zhang WH, Deneux-Tharaux C, Brocklehurst P, Juszczak E, Joslin M, Alexander S ( 2010). Effect of a collector bag for measurement of postpartum blood loss after vaginal delivery: cluster randomised trial in 13 European countries, EU- PHRATES Group. BMJ.

[16] Prasertcharoensuk W, Swadpanich U, Lumbiganon P ( 2000). Accuracy of the blood loss estimation in the third stage of labor. Int J Gynecol Obstet.

[17] Duthie SJ, Ven D, Yung GL, Guang DZ, Chan SY, Ma HK ( 1991). Discrepancy between laboratory determination and visual estimation of blood loss during normal delivery. Eur J Obstet GynecolReprod Biol.

[18] Stafford I, Dildy GA, Clark SL, Belfort MA ( 2008). Visually estimated and calculated blood loss in vaginal and cesarean delivery. Am J Obstet Gynecol.

[19] Phillips R, Friberg M, Lantz Cronqvist  M, Jonson CO, Prytz E ( 2020). Visual estimates of blood loss by medical laypeople: Effects of blood loss volume, victim gender, and perspective. PLOS ONE.

[20] Lertbunnaphong T, Lapthanapat N, Leetheeragul J, Hakularb P, Ownon A ( 2016). Postpartum blood loss: visual estimation versus objective quantification with a novel birthing drape. Singapore Med J.

[21] Anya SU, Onyekwulu FA, Onuora EC ( 2019). Comparison of visual estimation of intra-operative blood loss with haemoglobin estimation in patients undergoing caesarean section. Niger Postgrad Med J.

[22] Al Kadri  HM, Al Anazi  BK, Tamim HM ( 2011). Visual estimation versus gravimetric measurement of postpartum blood loss: a prospective cohort study. Arch Gynecol Obstet.

[23] Andrikopoulou M, D'Alton ME ( 2019). Postpartum hemorrhage: early identification challenges. Semin Perinatol.

[24] Blosser C, Smith A, Poole AT ( 2021). Quantification of Blood Loss Improves Detection of Postpartum Hemorrhage and Accuracy of Postpartum Hemorrhage Rates: A Retrospective Cohort Study. Cureus.

[25] Fedoruk K, Seligman KM, Carvalho B, Butwick AJ ( 2019). Assessing the Association Between Blood Loss and Postoperative Hemoglobin After Cesarean Delivery: A Prospective Study of 4 Blood Loss Measurement Modalities. Anesth Analg.

[26] he perfect fake blood recipes [Internet] 6 Steve Spangler Science [Accessed: 1 May 2021]. https://www.stevespanglerscience.com/wp-content/uploads/2016/09/Fake-Blood-Recipes-Steve-Spangler-Science.pdf.

[27] Skinner BF ( 1954). The science of learning and the art of teaching. Harvard Educational Review.

[28] Dildy GA, Paine AR, George NC, Velasco C ( 2004). Estimating blood loss: can teaching significantly improve visual estimation?. Obstet Gynecol.

[29] Cheerranichanunth P, Poolnoi P ( 2012). Using blood loss pictogram for visual blood loss estimation in cesarean section. J Med Assoc Thai.

[30] Sukprasert M, Choktanasiri W, Ayudhya NI, Promsonthi P, O-Prasertsawat P ( 2006). Increase accuracy of visual estimation of blood loss from education programme. J Med Assoc Thai.

[31] Toledo P, Eosakul ST, Goetz K, Wong CA, Grobman WA ( 2012). Decay in blood loss estimation skills after web-based didactic training. Simul Healthc.

[32] Wong CA, Scott S, Jones RL, Walzer J, Geller S ( 2016). The state of Illinois obstetric hemorrhage project: pre-project and post-training examination scores. J Matern Fetal Neonatal Med.

[33] Rothermel LD, Lipman JM ( 2016). Estimation of blood loss is inaccurate and unreliable. Surgery.

[34] Coviello E, Iqbal S, Kawakita T, Chornock R, Cheney M, Desale S, et al ( 2019). Effect of Implementing Quantitative Blood Loss Assessment at the Time of Delivery. Am J Perinatol.

